# Tumor-Infiltrating Immune Cell Landscapes in the Lymph Node Metastasis of Papillary Thyroid Cancer

**DOI:** 10.3390/curroncol30030200

**Published:** 2023-02-22

**Authors:** Md Amanullah, Meidie Pan, Kaining Lu, Xiaoqing Pan, Yan Lu, Dingcun Luo, Pengyuan Liu

**Affiliations:** 1 Key Laboratory of Precision Medicine in Diagnosis and Monitoring Research of Zhejiang Province, Sir Run Run Shaw Hospital and Institute of Translational Medicine, Zhejiang University School of Medicine, Hangzhou 310016, China; 2 Department of Surgical Oncology, Affiliated Hangzhou First People’s Hospital, Zhejiang University School of Medicine, Hangzhou 310006, China; 3 Department of Mathematics, Shanghai Normal University, Xuhui, Shanghai 200234, China; 4 Zhejiang Provincial Key Laboratory of Precision Diagnosis and Therapy for Major Gynecological Diseases, Women’s Hospital and Institute of Translational Medicine, Zhejiang University School of Medicine, Hangzhou 310006, China; 5 Cancer Center, Zhejiang University, Hangzhou 310013, China

**Keywords:** thyroid cancer, lymph node metastasis, tumor microenvironment, driver mutation, prognosis, immune cells

## Abstract

Regional lymph node metastasis (LNM) increases the risk of distant metastasis in papillary thyroid cancer (PTC) patients. However, it remains unclear how tumor cells in PTC patients with LNM evade immune system surveillance and proceed to colonize distant organs. Here, we comprehensively characterize the tumor-infiltrating immune cell landscape in PTC with LNM. LNM-related genes include multiple important soluble mediators such as CXCL6, IL37, MMP10, and COL11A1, along with genes involved in areas such as extracellular matrix organization and TLR regulation by endogenous ligands. In PTC without LNM, the tumor infiltration of activated dendritic cells and M0 macrophages showed increases from normal cells, but with yet greater increases and correspondingly worse prognosis in PTC with LNM. Conversely, the tumor infiltration of activated NK cells and eosinophils was decreased in PTC without LNM, as compared to normal cells, and yet further decreased in PTC with LNM, with such decreases associated with poor prognosis. We further demonstrate that mutations of driver genes in tumor cells influence the infiltration of surrounding immune cells in the tumor microenvironment (TME). Particularly, patients carrying TG mutations tend to show increased filtration of M2 macrophages and activated NK cells in the TME, whereas patients carrying HRAS mutations tend to show reduced filtration of M0 macrophages and show enhanced filtration of activated dendritic cells in the TME. These findings increase our understanding of the mechanisms of regional lymph node metastasis in PTC and its associated tumor microenvironment, potentially facilitating the development of personalized treatment regimens to combat immunotherapy failure.

## 1. Introduction

Globally, thyroid cancer (TC) is experiencing the greatest increase rate in incidence of any solid tumor cancer. According to the latest World Cancer Report, there were 586,000 new TC patients in 2020, which places TC as 9th among all cancers in global incidence, and as an increasing health issue over recent years [1]. Papillary thyroid cancer (PTC) is the most common type of TC, accounting for more than 90% of all newly diagnosed cases. Women are about three times more likely to be affected by PTC than men [2,3]. The overall prognosis of thyroid cancer is good, with a 10-year survival rate of more than 90% [4,5,6]. However, regional lymph node metastasis (LNM) occurs in approximately 40% of PTC patients [7,8]. LNM increases the risk of recurrence and distant metastasis for PTC [9,10]. The 10-year survival rate of PTC patients with recurrence and distant metastasis is then reduced to 24–76% [11,12]. However, it remains unclear how tumor cells in PTC patients with LNM evade immune system surveillance and proceed to colonize distant organs.

The tumor microenvironment (TME) represents the environment surrounding the tumor. It is comprised of blood vessels, immune cells, fibroblasts, signaling molecules, and the extracellular matrix [13,14,15]. Tumor cells are closely related to their surrounding immune cells, with which they constantly interact. These interactions are involved in all stages of cancer including tumor growth, cancer progression, and metastasis [14,16,17,18]. Immune characteristics in the TME are highly predictive of prognosis for cancer patients [19]. Immunotherapy medications can improve the ability of the immune system to detect and destroy tumor cells. Over recent years, a large number of patients have benefited from immunotherapy, including some patients with advanced cancers [20]. To make up for deficiencies in surgical and radiotherapy approaches [21], immunotherapy has been developed to attempt to change the tumor immune-related microenvironment in the treatment of thyroid cancer [22]. For example, the inhibitors sorafenib and lenvatinib were recently approved as immunotherapeutic agents for use in the treatment of progressive metastatic thyroid cancer [23].

BRAF or RAS activating mutations are prevalent in PTC, resulting in the up-regulation of CXCL10. CXCL10 is a chemokine which induces chemotaxis, promotes differentiation of immune cells, and causes tissue extravasation, thereby suppressing tumors [24]. However, there are also some conflicting studies that show that CXCL10 can stimulate the proliferation and invasion of thyroid cancer [25,26]. Patients with both LNM and wild-type BRAF showed only a moderate mortality risk, whereas patients with the BRAF V600E-activated mutations show strong mortality risk [27]. Another study showed CD8+ lymphocyte infiltration and COX2 expression as independent predictors of relapse risk in PTC patients [28]. However, in the presence of lymph node metastases at diagnosis, these markers were noted to have lost their prognostic significance. Therefore, comprehensive characterizations of the immune microenvironment in PTC, with and without LNM, may help clarify such issues and increase our understanding of the pathophysiology of LNM and its involvement in PTC tumor invasion and metastasis. In addition, assessments of various immune cell subtype compositions of PTC may also provide prognostic information and improve disease diagnosis and immunotherapy development for LNM patients at risk of recurrence and distant metastasis.

**Figure 1:**
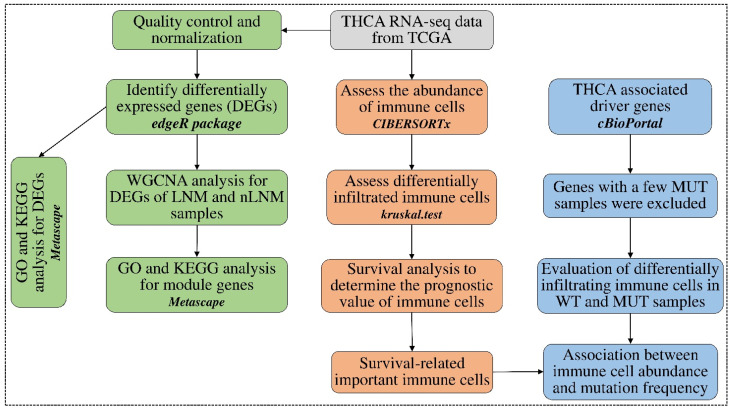
Flowchart of data analyses in the study. Three main analyses were conducted in the study: (i) The abundance of immune cells was estimated using CIBERSORTx. Differentially infiltrated immune cells between PTC groups were determined using the Kruskal–Wallis test. The association of immune cell abundances with patient survival was assessed using a univariate Cox regression model. (ii) Differentially expressed genes were identified between different PTC groups using edgeR. Immune cell-related biomarkers were identified using WGCNA analysis. (iii) The relationship between immune cell abundances and driver gene mutations in PTC was evaluated.

In this study, we conducted comprehensive characterization of tumor-infiltrating immune cells in PTC specimens, with and without LNM, from The Cancer Genome Atlas (TCGA) (Figure 1). We identified genes consistently deregulated in PTC with LNM, and systematically assessed the association of the infiltration of various immune cell subsets with LNM status, prognosis information, and the mutational status of driver genes in PTC. Furthermore, we identified gene co-expression modules associated with prognosis-related immune cell subsets. These findings provide novel insights into the mechanisms of regional lymph node metastasis in PTC and its associated tumor immune microenvironment. Immune cell-related biomarkers are also identified for predicting the risk of recurrence and distant metastasis in PTC with LNM.

## 2. Materials and Methods

### 2.1. Data Sources and Data Pre-Processing

The RNA-seq data, in the format of read counts (hg38) and corresponding clinical data of PTC specimens, were downloaded from the TCGA Genomic Data Commons (GDC) database. Tumor samples were classified into PTC with LNM and those without LNM, based on the TNM tumor staging criteria (https://www.cancer.org/cancer/thyroid-cancer/detection-diagnosis-staging/staging.html, accessed on 14 June 2021). Briefly, according to tumor stage (T1-T4), N stage (N0-N1), and metastases stage (M0-M1), T1-4N1M0 was defined as PTC with LNM (n = 131), while T1-4N0M0 was defined as PTC without LNM (n = 145). Other PTC specimens that did not belong to these two categories were not included in this study (Supplementary Table S1). Additionally, 58 normal samples adjacent to PTC tumor tissues were also included for analyzing tumor-infiltering immune microenvironments. Genes with 0 reads counted in more than 80% of all samples were excluded, leaving 36,850 remaining genes. In addition, since a single gene can produce multiple transcripts, we selected the one with the largest median expression of multiple transcripts from the same gene. A total of 33,203 genes were retained under this process for subsequent analysis (Figure 1).

### 2.2. Estimation of the Abundance of Tumor-Infiltering Immune Cells

CIBERSORTx, a recently developed machine-learning method, was applied to characterize tumor-infiltering immune cell landscapes in PTC tumor tissues and adjacent normal tissues [29]. The advantage of CIBERSORTx over its predecessor CIBERSORT [30] is its ability to accurately estimate absolute rather than relative abundances for each lymphocyte within bulk expression data. The LM22 reference signature was used to estimate the composition of 22 lymphocytes in PTC tumor tissues and adjacent normal tissues. The LM22 signature matrix contains 547 genes, of which 514 have available expression data in the PTC dataset. Gene expression data were normalized with CPM (counts per million) and uploaded to the CIBERSORTx official website. The run mode was selected as bulk-mode, with LM22 signature matrix and 500 permutations also selected. The B-mode batch correction method was enabled while the quantile normalization was disabled when running RNAs-seq samples. Samples with a *p*-value > 0.05 were excluded, as their immune cell compositions were considered to have not been reliably estimated.

### 2.3. Identification of Differential Abundances of Immune Cells between PTC Groups

Immune cells with a median abundance close to zero were excluded from the study. The abundances of immune cell subtypes were not normally distributed, as indicated by the Shapiro–Wilk normality test. Therefore, the Kruskal–Wallis rank-sum test was applied to identify differential abundance of infiltrating immune cells between normal, PTC without LNM, and PTC with LNM groups. Mean differences in the immune cell abundances between groups were considered as significant upon a *p*-value of <0.05.

### 2.4. Identifying Survival-Related Tumor-Infiltrating Immune Cells

The association of the abundance of each of the immune cell subsets with survival was assessed using univariate Cox proportional hazards regression analysis. Clinical and follow-up data such as overall survival (OS) and progression-free survival (PFS) of PTC patients were collected from TCGA. The *p*-value and hazard ratio with a 95% confidence interval were estimated. In addition, Kaplan–Meier curve analysis was used to visualize the survival differences between groups of samples according to their median abundances. The log-rank test was used to assess the significant differences in survival between groups. All survival analyses were implemented in the “survival” R package.

### 2.5. Detecting Differentially Expressed Genes between PTC Groups

Differentially expressed genes (DEGs) between tumor tissues from PTC with LNM (n = 131) and normal tissues (n = 58), between tumor tissues from PTC without LNM (n = 145) and normal tissues, and between PTC with and without LNM were detected using the “edgeR” R package [31]. The trimmed mean of M-values (TMM) method was used to calculate the normalization factors to scale the raw library sizes. Moreover, a robust quasi-likelihood generalized linear model was fitted to estimate the prior dispersion distributions. Significant genes were selected when adjusted (Benjamin and Hochberg method) *p*-value < 0.05 and |log_2_FC| > 1.5. Heatmaps of the three sets of samples were drawn using the “ComplexHeatmap” R package. DEGs from each comparison were uploaded to the Metascape portal to explore gene ontology (GO) terms and the KEGG pathways enriched for these DEGs [32].

### 2.6. Discovering Immune-Related Genes Using WGCNA Analysis

Weighted co-expression network analysis (WGCNA) was used to find the co-expression modules highly correlated with the abundances of different types of immune cells [33]. DEGs between PTC with and without LNM were used to construct a gene co-expression network. Most biologically relevant networks are known to be scale free, consisting of many lesser connected genes and a small number of highly connected hub genes [34]. To identify a scale-free gene co-expression network, different thresholds of power varying from 1 to 20 were examined. As a result, a power *β* = 3 with *R*^2^ = 0.90 (the fitting regression index) was determined as a soft threshold that satisfied the scale-free topology of the gene co-expression network. Next, the network adjacency matrix was calculated using the gene expression and *β* = 3, which was subsequently converted into the topological overlap matrix (TOM) and corresponding dissimilarity matrix. Hierarchical cluster analysis was implemented with the average linkage method for clustering genes within the module. The minimum number of genes in a module was set to 30 to avoid over repetition of analysis of highly similar genes. The gene network was presented in a heatmap based on TOM dissimilarity with its cluster dendrogram. The module eigengene (ME), the first principal component in a module, was considered to provide a strong and appropriate representation of the genes in the module. Correlations between ME and the abundance of each type of immune cells were then utilized to identify the immune cell-associated targeted modules.

### 2.7. Correlation between Immune Cell Abundances and Gene Mutation Profiles

We also explored the correlation of driver gene mutations with immune cell abundances in PTC. A recent study has reported that mutations in 24 genes were causally implicated in PTC [35]. The mutational status of these driver genes was determined using the cBioportal web tool (http://cbioportal.org, accessed on 1 July 2021). Genes with few mutations were excluded, leaving 20 genes for subsequent analysis. The Wilcoxon test was used to identify the differential infiltration of immune cells between wild-type (WT) and mutant (MUT) for the driver genes in PTC. Survival differences between the WT and MUT groups were also assessed using Kaplan–Meier curve analysis with a log-rank test. Furthermore, correlation analysis was carried out between immune cell abundances and mutational frequencies of these driver genes in PTC. Somatic mutation (SNPs and small INDELs) frequency datasets for PTC samples were obtained from UCSC Xena (https://gdc.xenahubs.net, accessed on 1 July 2021), where mutation frequencies were calculated by the ratio of alternative allele counts to total sequence depth. Similarly, the correlation between immune cell abundances and mutation frequencies of driver genes was adjusted by the tumor purity of PTC. The tumor purity data for PTC, which were calculated by the most commonly used algorithm ESTIMATE, were obtained from a previous study [36].

## 3. Results

### 3.1. Tumor-Infiltrating Immune Cell Landscape in PTC

**Figure 2:**
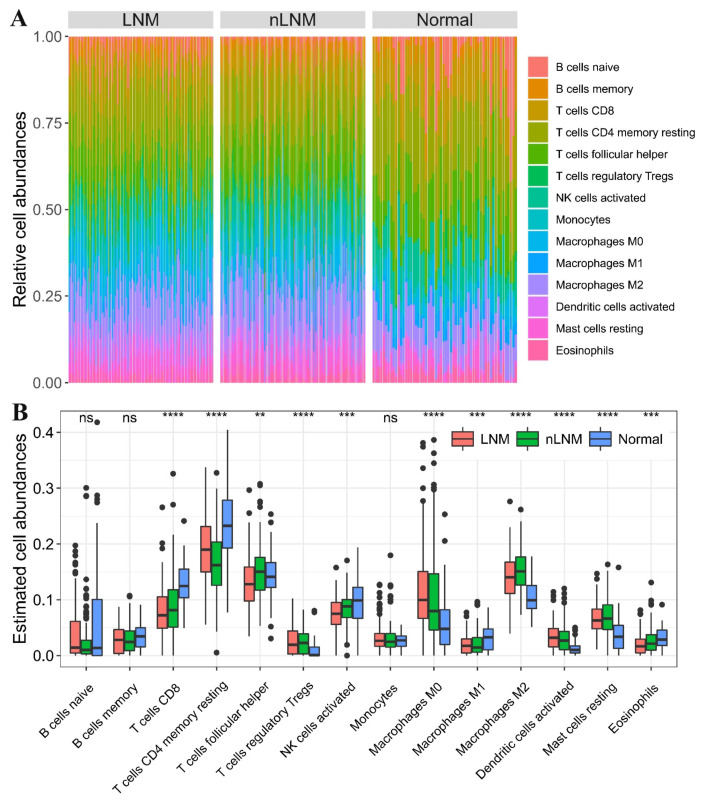
Tumor-infiltrating immune cell abundances in PTC. (**A**) Measurement of immune cell abundances in different PTC groups using CIBERSORTx. Eight immune cell subtypes with a median abundance close to zero were excluded from subsequent comparisons. (**B**) Comparison of infiltrating immune cells between different PTC groups. Differences in immune cell abundance between three PTC groups were assessed by the Kruskal–Wallis rank-sum test. ns: *p* > 0.05, **: *p* ≤ 0.01, ***: *p* ≤ 0.001, ****: *p* ≤ 0.0001. LNM and nLNM represent PTC with and without LNM, respectively. ‘Normal’ represents normal thyroid tissue adjacent to the PTC tumor.

We analyzed a total of 334 PTC specimens, comprised of 145 tumor tissues from PTC without LNM, 131 tumor tissues from PTC with LNM, and 58 adjacent normal tissues. The abundances of 22 types of immune cells in these samples were estimated using CIBERSORTx. Eight types of immune cells were present in these samples at extremely low abundance (levels close to zero). Therefore, we focused on the remaining 14 types of immune cells for subsequent analyses. The relative abundances of each type of immune cells for these samples are shown in the stacked bar graphs (Figure 2A), suggesting a large variability in immune cell composition between PTC samples. T cells were made up of approximately 40% of immune cells, with resting memory CD4 T cells and follicular helper T cells accounting for the majority of these, and ‘regulatory T cells’ (Tregs) accounting for the smallest proportion. Macrophages constituted approximately 24% of immune cells, with ‘M2 macrophages’ being the predominant subtype. Activated NK cells and mast cells accounted for 8.4% and 6.0% of immune cells, respectively. B cells and activated dendritic cells accounted for 4% and 2.4% of immune cells, respectively. The other rare immune cell subtypes together accounted for the remaining immune cells (approximately 15%).

The average abundance of each type of immune cell was then compared between different PTC groups (Figure 2B). As a result, 11 lymphocytes showed significantly different abundances between different PTC groups (*p* < 0.05). Overall, the abundances of CD8 T cells, resting memory CD4 T cells, activated NK cells, M1 macrophages, and eosinophils were significantly reduced in PTC tumor tissues compared with adjacent normal tissues. Similarly, the abundances of regulatory T cells, M0 macrophages, M2 macrophages, activated dendritic cells, and resting mast cells were significantly increased in PTC tumor tissues. Notably, the abundances of CD8 T cells, activated NK cells, and eosinophils were significantly decreased in PTC without LNM compared with normal thyroid tissues, whereas all of three aspects were further reduced in PTC with LNM (*p* < 0.05). Conversely, the abundances of M0 macrophages and activated dendritic cells were significantly increased in PTC without LNM, as compared with normal thyroid tissues, and were further increased in PTC with LNM (*p* < 0.05) (Figure 2B). Taken together, these results reveal marked heterogeneity of tumor-infiltrating lymphocytes in PTC tumors, as well as distinct tumor-infiltering immune cell patterns for PTC occurring with and without LNM.

### 3.2. Tumor-Infiltrating Immune Cells Associated with Prognosis

We used a univariate Cox regression model to analyze the association of immune cell abundance with PTC prognosis. In this, we identified four types of immune cells associated with PTC prognosis (Table 1). Specifically, the abundance of activated NK cells (*p* = 0.003, *HR* = 0.892) was significantly associated with longer overall survival, while M0 macrophages (*p* = 0.04, *HR* = 1.018) and activated dendritic cells (*p* = 0.033, *HR* = 1.077) were significantly correlated with shorter overall survival. The abundances of activated NK cells (*p* = 0.039, *HR* = 0.963) and eosinophils (*p* = 0.023, *HR* = 0.933) were significantly correlated with longer progression-free survival, while the abundance of M0 macrophages (*p* = 0.018, *HR* = 1.013) was significantly correlated with shorter progression-free survival. In addition, we divided the PTC samples into two groups according to the median abundance of each of these four immune cell subsets, and then carried out Kaplan–Meier curve analysis to visualize survival differences between the two groups. In comparing the four types of immune cells, the survival differences and other trends largely remained between the two groups based on their median abundances (Supplementary Figure S1). These results suggest that tumor-infiltrating immune cells are valuable prognostic biomarkers and may also play important roles in tumor recurrence and distant metastasis for PTC.

### 3.3. Genes Associated with LNM

**Figure 3:**
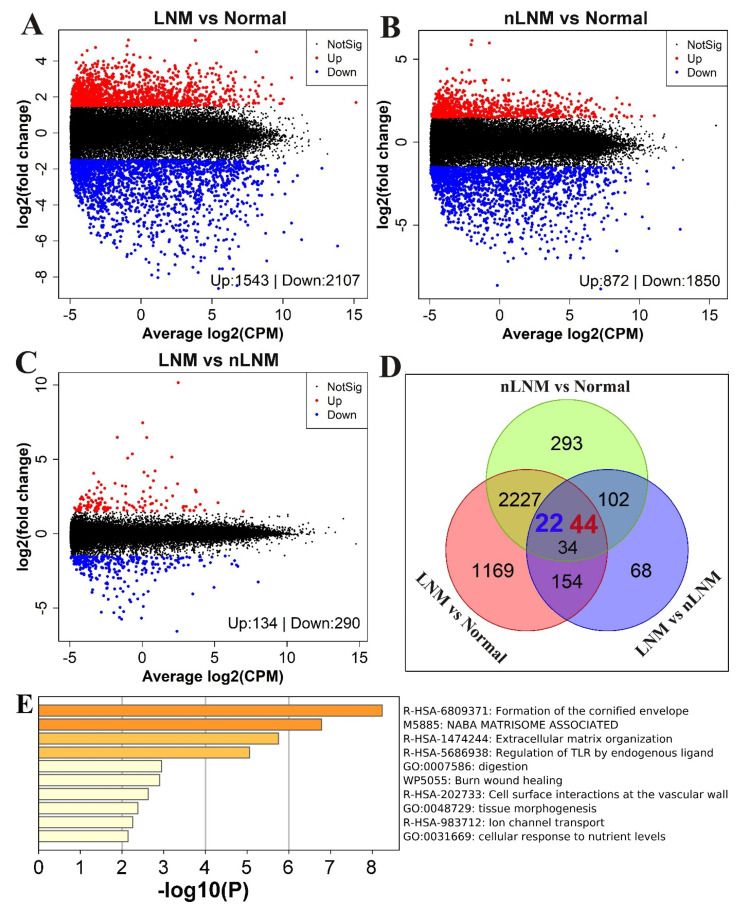
Differentially expressed genes between PTC with and without LNM. (**A**–**C**) Volcano plot showing the log2-fold changes (log_2_FC) in gene expression and their associated log2-mean expression (log_2_CPM) between normal and LNM, between normal and nLNM, and between LNM and nLNM. Red dots mark the up-regulated genes with log_2_FC > 1.5 and FDR < 0.05 and blue dots mark the down-regulated genes with log_2_FC < −1.5 and FDR < 0.05, in each comparison. (**D**) Venn diagram of the DEGs genes identified in the three comparisons. A total of 100 DEGs were detected among 3 comparisons. Among them, 44 DEGs were up-regulated in PTC without LNM compared to normal thyroid tissues and were further up-regulated in PTC with LNM compared to PTC without LNM. Conversely, 22 DEGs were down-regulated in PTC without LNM compared to normal thyroid tissues and were further down-regulated in PTC with LNM compared to PTC without LNM. (**E**) Pathway enrichment analysis for these 66 DEGs (22 down-regulated and 44 up-regulated).

We next attempted to identify genes that could be potentially associated with LNM in PTC samples. Differential expression analyses were performed between PTC with LNM and adjacent normal tissues (LNM vs. Normal), between PTC without LNM and adjacent normal tissues (nLNM vs. Normal), and between PTC with and without LNM (LNM vs. nLNM), respectively. This resulted in 3650, 2722, and 424 DEGs identified in the group comparisons of LNM vs. Normal, nLNM vs. Normal, and LNM vs. nLNM, respectively (Figure 3A–C). The number of DEG overlaps between these three comparisons and is shown in a Venn diagram (Figure 3D). Overall, PTC with and without LNM and the adjacent normal tissues all showed distinct gene expression patterns (Supplementary Figure S2A).

In the comparison of LNM vs. Normal, 1543 and 2107 DEGs were up- and down-regulated, respectively. GO terms enriched for these DEGs mainly included regulation of hormone levels, chemotaxis, wound response, and the positive regulation of the MAPK cascade. The KEGG pathways enriched for these DEGs were mainly those involved in extracellular matrix organization, GPCR ligand binding, regulation of insulin-like growth factor (IGF) transport, and up-take by insulin-like growth factor binding proteins (IGFBPs) (Supplementary Figure S3A).

In the comparison of nLNM vs. Normal, 872 and 1850 DEGs were up- and down-regulated, respectively. GO terms enriched for these DEGs mainly included cell junction organization, wound response, positive regulation of the MAPK cascade, and the regulation of cell adhesion. KEGG pathways enriched for these DEGs were those mainly involved in GPCR ligand binding, regulation of insulin-like growth factor (IGF) transport, up-take by insulin-like growth factor binding proteins (IGFBPs), class A/1 (rhodopsin-like receptors), and extracellular matrix organization (Supplementary Figure S3B).

In the comparison of LNM vs. nLNM, 134 and 290 DEGs were up- and down-regulated in LNM, respectively (Supplementary Figure S3C). Surprisingly, a total of 14 of the top 20 GO terms and pathways were unique for the DEGs in the comparison of LNM vs. nLNM (Supplementary Figure S2B). In particular, 44 DEGs were up-regulated in PTC without LNM, as compared to normal thyroid tissues, and these DEGs were further up-regulated in PTC with LNM compared to PTC without LNM, of which multiple important soluble mediators, such as CXCL6, IL36G, IL37, MMP10, MMP13, and COL11A1, were detected (Figure 3D). Conversely, 22 DEGs were down-regulated in PTC without LNM compared to normal thyroid tissues, these being yet further down-regulated in PTC with LNM compared to PTC without LNM. The degree of dysregulation of these DEGs appeared to correlate with the aggressiveness of LNM in PTC. GO terms and pathways enriched for these 66 DEGs were shown to be mainly those involved in extracellular matrix organization, regulation of TLR by endogenous ligands, cell surface interactions at the vascular wall, ion channel transport, and other related areas (Figure 3E). Taken together, these results suggest that dysregulation of many genes, such as multiple important solubility mediators, may lead to the progression of PTC to regional lymph node metastases.

### 3.4. Co-Expression Module Analysis to Identify Immune Cell-Related Genes

**Figure 4:**
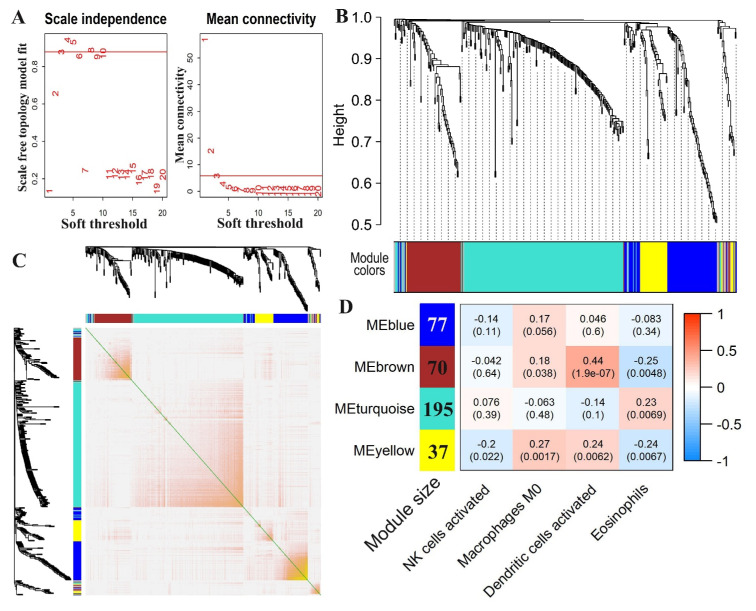
Co-expression networks constructed by WGCNA. (**A**) Selection of appropriate soft threshold for network topology analysis. (**B**) Hierarchical clustering dendrogram of co-expressed genes identified in the module. The dendrogram was created via unsupervised hierarchical clustering of genes. Each module was presented in a specific color. (**C**) Heatmap of co-expression gene modules based on TOM dissimilarity. The level of similarity is represented by the intensity of the yellow in the heatmap, where the yellow-yellow color indicates an increase in similarity. (**D**) Heatmap of correlations between immune cell abundances and module eigengenes. An eigengene was defined as the first principal component of the expression matrix of the corresponding module, which could be considered a representation of the gene expression profiles within a module. Each row corresponds to a module and each column corresponds to the abundance of one subtype of immune cells. Each cell has a correlation coefficient and its associated *p*-value in parentheses. Light blue represents negative correlation and light red represents positive correlation. Dark colors in the heatmap represent genes that are highly correlated in the module. The size of each module is shown in the left column of the heatmap.

A total of 424 DEGs between PTC with and without LNM were then used to construct a co-expression network. To obtain an approximate scale-free topology, a scale-free soft thresholding parameter *β* = 3 was selected, yielding a fit index *R*^2^ of 0.9 (Figure 4A). The dynamic cutting tree identified four modules, each of which contained a set of functionally related genes with similar co-expression patterns (Figure 4B). Gene network heatmaps were created based on the TOM dissimilarity and their cluster dendrogram (Figure 4C). Module–trait association was performed by analyzing the correlations between immune cell abundance and the module eigengene, which is a representation of the gene expression profiles in a module. As a result, four types of survival-related immune cells were identified to be associated with these gene modules (Figure 4D). The highest positive correlation was observed between brown module and dendritic cell activation (r = 0.44, *p*-value = 1.90 × 10^−7^), while the highest negative correlation was observed between brown module and eosinophil cells (r = −0.25, *p*-value = 0.0048) (Figure 4D). Genes in the brown module were enriched towards several immune response pathways including humoral immune response (Supplementary Figure S4A). In addition, the turquoise gene module was positively correlated with the abundance of eosinophils in PTC tumors. Genes in this module were enriched for the lipid metabolism (Figure 4D and Supplementary Figure S4B). The yellow module was positively associated with M0 macrophages and activated dendritic cells and negatively associated with activated NK cells and eosinophils. Genes in this module were enriched for adaptive immune responses (Figure 4D and Supplementary Figure S4C).

### 3.5. Immune Cell Abundance Associated with Driver Gene Mutation

**Figure 5:**
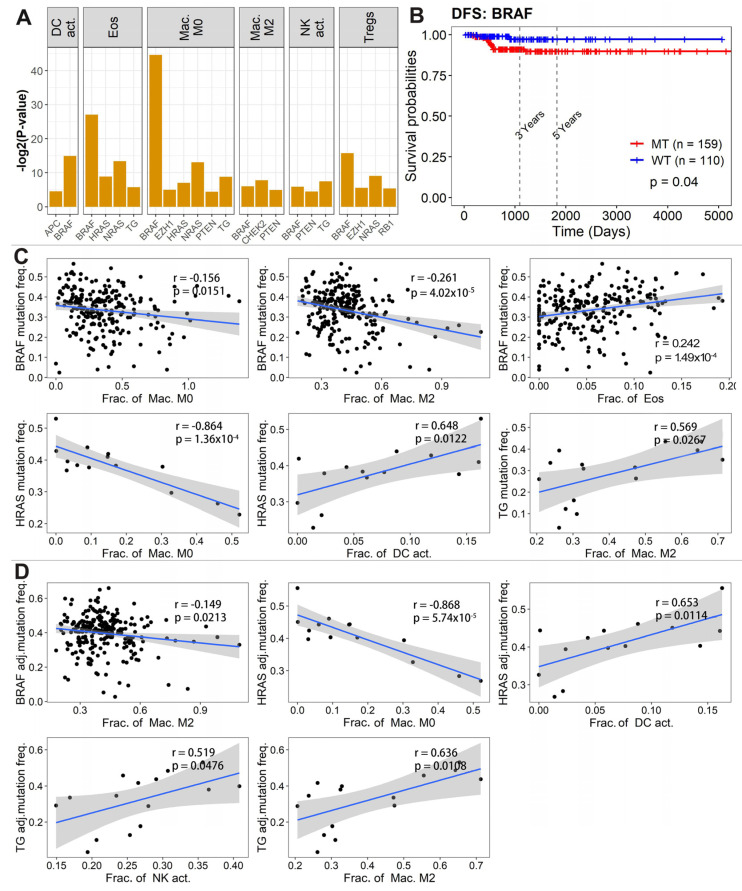
Relationship between immune cell abundances and driver gene mutations. (**A**) Differences in the abundances of immune cells between wild-type and mutants of driver genes in PTC, as assessed by the Wilcoxon test with *p*-value < 0.05. (**B**) Kaplan–Meier survival curve of patients carrying BRAF mutation and wild-type (disease-free survival). (**C**) Correlation between immune cell abundances and mutation frequencies of BRAF, HRAS, and TG genes. The results of other genes were not presented due to their very low levels of somatic mutation. (**D**) Correlation between immune cell abundances and adjusted mutation frequencies of BRAF, HRAS, and TG genes. Somatic mutation frequencies were adjusted by tumor purity, which was estimated using the ESTIMATE algorithm.

Finally, we assessed the association of immune cell abundances with the mutational statuses of the 24 driver genes that had been previously detected in the tumor genome sequencing of PTC [35]. The differences in the abundances of 11 types of immune cells between mutant and wild-type of these driver genes were examined using a Wilcoxon test (Figure 5A). The abundances of six types of immune cells were significantly different between the mutant and wild-type of nine driver genes (BRAF, HRAS, NRAS, APC, PTEN, TG, CHEK2, RB, and EZH1) (Figure 5A and Supplementary Table S2). Among these nine driver genes, the mutational status of BRAF was also associated with the disease-free survival of PTC and patients with the BRAF mutation tended to have a worse prognosis (Figure 5B). In addition to mutational status, we further evaluated the association of immune cell abundances with mutation frequencies of these driver genes (Figure 5C). We observed that the mutation frequencies of BRAF, HRAS, and TG genes were correlated with multiple immune cell subtypes. Specifically, the mutation frequency of BRAF was negatively correlated with M0 macrophages (r = −0.156, *p* = 0.015) and M2 macrophages (r = −0.0261, *p* = 4.02 × 10^−5^) and positively correlated with eosinophils (r = 0.242, *p* = 1.490 × 10^−4^). The mutation frequency of HRAS was negatively associated with M0 macrophages (r = −0.846, *p* = 1.36 × 10^−4^) and positively associated with activated dendritic cells (r = 0.648, *p* = 0.012). The mutation frequency of TG genes was positively associated with M2 macrophages (r = 0.569, *p* = 0.027) (Figure 5C). To rule out the potential confounding effect of tumor purity, correlation analysis was further performed between immune cell abundances and the adjusted mutation frequencies of genes by tumor purity. Overall, the relationships between immune cell abundances and tumor purity-adjusted mutation frequencies were largely consistent with the unadjusted ones (Figure 5D). Taken together, these results showed that immune cell type abundances differ between PTC tumors with or without these driver mutations, suggesting that tumor cells and surrounding immune cells are closely related and potentially interact with each other.

## 4. Discussion

A considerable portion of patients with PTC also develop regional lymph node metastasis, which then increases the risk of later tumor recurrence and more distant metastasis [7,8,9,10]. Immunotherapy has emerged as a promising treatment option for advanced thyroid cancer and an attractive alternative option to more routine treatment that includes a number of difficulties, surgical complications [21], and which lacks a high degree of success [37,38]. However, a better understanding of TME in thyroid cancer is critical for improving immunotherapy efficacy and patient outcomes. Previous studies have reported that some immune cells are expressed in thyroid cancer, including T cells, macrophages, dendritic cells, and Tregs [39,40]. Their various roles in the TME, such as in tumor growth and invasion (T cells and macrophages), and in immune evasion (Tregs, T cells, and dendritic cells), have been already explored in PTC to a certain extent [40,41]. However, despite different types of immune cells having more general associations with tumor progression and metastasis, previous studies have lacked any specific focus on the role of immune cells related to LNM in PTC [42,43]. In addition, these studies had estimated relative cellular abundance rather than absolute cellular abundance from bulk gene expression data [42,43]. The recently developed CIBERSORTx enables the estimation of absolute cell abundance in tumor tissues from bulk RNA-seq data [29], allowing for better characterization of the relationship between cell abundance and clinicopathological features such as prognosis. In this study, we comprehensively characterize the tumor-infiltrating immune cell landscapes in PTC with LNM using CIBERSORTx. This has led to the uncovering of several novel characteristics of TME of potentially great significance towards the understanding of the molecular mechanism of LNM in PTC.

Firstly, we observed significantly different levels of infiltration of immune cells between PTC with and without LNM. Notably, the abundances of CD8 T cells, activated NK cells, and eosinophils were significantly reduced in PTC without LNM compared with normal thyroid tissues, and all three aspects were further depleted in PTC with LNM. Conversely, the abundances of M0 macrophages and activated dendritic cells were significantly increased in PTC without LNM compared with normal thyroid tissues, and these were also further increased in PTC with LNM. Of these, the abundances of activated NK cells and eosinophils were associated with favorable prognosis, while the abundances of activated dendritic cells and M0 macrophages were associated with poor prognosis. These data suggest that the infiltration of these immune cell subsets is a valuable prognostic biomarker for PTC and may also play an important role in promoting PTC tumor cell invasion into regional lymph nodes.

NK cells are a type of cytotoxic lymphocyte critical to the innate immune system. They show strong cytolytic function against cancer cells in TME [44]. The infiltration of NK cells in early stages of PTC is higher than that in advanced stages [45]. In the present study, the infiltration of NK cells in PTC with LNM is reduced compared with PTC without LNM. More NK cell infiltration in PTC tends to predict better prognosis. Eosinophils are a type of disease-fighting white blood cells which are noted to infiltrate multiple tumors. They can synthesize and secrete many molecules, including unique granule proteins, which may act to kill tumor cells [46]. Our analysis indicated that eosinophils are significantly reduced in PTC with LNM compared with PTC without LNM and that PTC patients with more eosinophil infiltration had distinctly better prognosis. Naïve macrophages (i.e., M0) were also noted as significantly increased in PTC with LNM compared with PTC without LNM. Previously, melanoma patients with localized tumors with M0 macrophage-enriched phenotypes had been seen to exhibit shorter survival [47]. This strongly corresponds with our results showing that increased M0 macrophage infiltration is associated with poor prognosis in PTC patients. Dendritic cells are a diverse group of specialized antigen-presenting cells (APC) that play key roles in the initiation and regulation of innate and adaptive immune responses [48]. A dendritic cell subset has the ability to transport antigens from the tumor towards the draining lymph nodes. Such migrating dendritic cells are the only APC subset capable of inducing the strong activation and proliferation of CD8+ T cells ex vivo [49,50]. In our analysis, activated dendritic cells increased the filtration of PTC with LNM compared to PTC without LNM. This may lead to the understanding that the increased filtration of dendritic cells in PTC with LNM may be a response to regional lymph node metastasis, the purpose of which is to deliver antigens to drain lymph nodes and to enhance the activation and proliferation of CD8+ T cells. Future studies are required to confirm this finding and investigate the mechanisms of these altered immune cell subsets in the regional lymph node metastasis of PTC tumors.

Secondly, we identified genes differentially expressed between PTC with and without LNM. Among them, 66 DEGs were not only deregulated in PTC compared with normal thyroid tissues, but also were consistently deregulated in PTC with LNM compared with PTC without LNM. These included multiple important soluble mediators such as CXCL6, IL36G, IL37, MMP10, MMP13, and COL11A1. Of note, CXCL6 was up-regulated in PTC without LNM compared with normal thyroid tissues, and further up-regulated in PTC with LNM compared with PTC without LNM. CXCL6 is known to act as a pro-angiogenic factor and noted to promote the growth and metastasis of various cancers, including melanomas [51], colon cancer [52], and lung cancer [53]. Other LNM-related genes are highly enriched towards functions including extracellular matrix organization, regulation of TLR by endogenous ligands, cell surface interactions at the vascular wall, ion channel transport, and other similar aspects. Further study of these LNM-related genes not only increases our understanding of the mechanisms of local lymph node metastasis, but also provides new biomarkers for predicting LNM in PTC.

Thirdly, we correlated the abundance of these prognosis-associated immune cells with the co-expression of gene modules. A gene module is a set of highly interrelated genes that tend to share similar gene regulations and biological functions. Interestingly, these prognosis-associated immune cells were highly correlated with several gene modules. For example, the brown module showed significant positive correlation with the abundance of activated dendritic cells in PTC with LNM. The genes in this module were enriched for several immune response pathways including humoral immune response. The turquoise gene module showed significant positive correlation with the abundance of eosinophils in PTC with LNM. Genes in this module were enriched for the lipid metabolism. Further investigation of these gene modules may shed light on the mechanisms by which gene modules regulate the proliferation and activation of these immune cell subsets in regional lymph node metastasis in PTC.

Fourthly, we demonstrated that driver gene mutations in tumor cells influence the filtration of immune cells in the TME. Tumor cells constantly interact with their surrounding immune cells. Such interactions are involved in all stages of cancer, including tumor growth, progression, and metastasis [14,16,17,18]. Tumor gene mutation plays an important role in tumor-related lymphocytic infiltration [54]. Our analysis showed that both mutational status and frequency of driver genes were associated with the abundances of immune cells in PTC. For example, patients carrying TG mutations tended to show increased filtration of M2 macrophages and activated NK cells in the TME, and patients carrying HRAS mutations tended to show reduced filtration of M0 macrophages, but enhanced filtration of activated dendritic cells in the TME. These data suggest that tumor driver mutations and the immune microenvironment appear to co-evolve to mutually promote each other, in which driver mutations may facilitate the formation of a pro-tumor immune microenvironment, and the resulting tumor microenvironment may in turn promote the evolution and progression of tumor cells in PTC. Understanding the crosstalk between tumor cells and the immune microenvironment in lymph node metastasis will hopefully facilitate the development of new combination therapies that can overcome tumor immune evasion mechanisms and improve the clinical efficacy of current immunotherapies.

Several caveats of this study should be acknowledged. Firstly, most cases of PTC in TCGA are well-differentiated thyroid cancers. Other histologic types of PTC are very few and remain insufficient for statistical analysis and comparison of the tumor immune microenvironment between LNM and non-LNM PTC. However, it remains of great significance to study the tumor immune microenvironment of other rarer and highly malignant tumors associated with LNM, such as poorly differentiated thyroid cancer, anaplastic thyroid cancer, and medullary thyroid cancer, in larger clinical cohorts in the future. Secondly, although several new characteristics of TME related to LNM have been revealed, additional validation assays such as immunohistochemistry and flow cytometry are needed to confirm these new findings in future studies. Finally, the potential diagnostic and prognostic biomarkers related to LNM immunity found in this study need to be verified using a larger independent sample prior to any clinical application.

## 5. Conclusions

Here, we have unveiled distinct tumor-infiltrating immune cell abundance and expression patterns between PTC with and without LNM. Compared with PTC without LNM, the tumor infiltration of activated dendritic cells and M0 macrophages are increased and associated with poor prognosis, while the tumor infiltration of activated NK cells and eosinophils are decreased, with such decreases also associated with poor prognosis in PTC with LNM. LNM-related genes are mainly involved in extracellular matrix organization, regulation of TLR by endogenous ligands, and cell surface interactions at the vascular wall. Mutations of driver genes in tumor cells are associated with the infiltration of surrounding immune cells in the TME. These findings increase our understanding of the mechanisms of regional lymph node metastasis in PTC and its associated tumor immune microenvironment and may facilitate the development of personalized treatment regimens to combat immunotherapy failure.

## Figures and Tables

**Table 1:** Abundances of immune cell subsets associated with prognosis of PTC patients.

	Immune Cells	Log Test *p*	HR	Upper Limit	Lower Limit	Z Score
OS	NK cells activated	0.003	0.892	0.969	0.821	−2.697
	Macrophages M0	0.040	1.018	1.034	1.002	2.233
	Dendritic cells activated	0.033	1.077	1.149	1.009	2.227
PFS	NK cells activated	0.039	0.963	1.000	0.927	−1.970
	Macrophages M0	0.018	1.013	1.024	1.003	2.488
	Eosinophils	0.023	0.933	0.993	0.875	−2.162

OS: overall survival; PFS: progression-free survival; HR: hazard ratio.

## Data Availability

The RNA-seq data used in the study were downloaded from the TCGA-THCA GDC database (https://portal.gdc.cancer.gov, accessed on 14 June 2021). The authors declare that all the other data supporting the findings of this study are available within the article.

## Supplementary Materials

The following supporting information can be downloaded at: https://www.mdpi.com/article/10.3390/curroncol30030200/s1, Table S1 Descriptive summary of thyroid carcinoma patients with and without LNM; Table S2 Differential abundances of immune cells between wild-type and mutant samples; Figure S1. Kaplan–Meier analysis of PTC’s survival based on abundances of immune cells. (A) Overall survival (OS). (B) Progression-free survival (PFS). PTC patients were divided into two groups according to their median levels of immune cell abundance. Difference in survival time between two groups was assessed using a log-rank test. The red and blue colors indicate the high and low abundance of immune cells, respectively. The two vertical gray dashed lines indicate the patient’s 3-year and 5-year survival times, respectively. PTC samples with p-values > 0.05 were excluded based on CIBERSORTx estimates of tumor-infiltrating immune cell abundance, as their immune cell composition was not considered reliably estimated. Therefore, different numbers of PTCs were able to be analyzed in each KM analysis. Figure S2. Heatmap of differentially expressed genes between PTC groups. (A) The heatmap shows the expression patterns of 300 highly DEGs in three groups (PTC tumors with and without LNM, and adjacent normal tissues), of which 100 highly DEGs were selected from each pair of comparisons. Each column represents a sample, and each row represents a gene. The expression level of each gene in a single sample is depicted according to the color scale. (B) Venn diagram showing the number of highly enriched pathways overlapping between the 3 pairs of comparisons, of which 20 highly enriched pathways were selected from each comparison pair. Figure S3. Functional enrichment analysis of differentially expressed genes between PTC groups. (**A**) Top 20 GO and KEGG pathways enriched for DEGs between LNM and normal. (**B**) Top 20 GO and KEGG pathways enriched for DEGs between nLNM and normal. (**C**) Top 20 GO and KEGG pathways enriched for DEGs between LNM and nLNM. Enrichment analysis was performed using the Metascape portal. The *x*-axis corresponds to −log10 *p*-values of enrichment, while the *y*-axis indicates enriched pathways. The color density indicates the significance level of a given pathway. Figure S4. GO and pathway enrichment analysis of three gene co-expression modules. Enrichment analysis of GO terms and pathways conducted for the gene modules correlated with survival-related immune cell subsets. (**A**) Brown module that showed the highest positive correlation with dendritic cells activated, and the highest negative correlation with eosinophils. (**B**) Turquoise module that was positively correlated with the abundance of eosinophils. (**C**) Yellow module that was positively correlated with M0 macrophages and activated dendritic cells and negatively correlated with activated NK cells and eosinophils. The *x*-axis corresponds to −log10 *p*-values of enrichment, while the *y*-axis indicates enriched GO and pathways.

## Abbreviations

CPM	Counts per million
DEGs	Differentially expressed genes
DFS	Disease-free survival
DSS	Disease-specific survival
HR	Hazard ratio
IGF	Insulin-like growth factor
IGFBPs	Insulin-like growth factor binding proteins
LNM	Lymph node metastasis
ME	Module eigengene
OS	Overall survival
PFS	Progression-free survival
PTC	Papillary thyroid cancer
TC	Thyroid cancer
TCGA	The Cancer Genome Atlas
TME	Tumor microenvironment
TOM	Topological overlap matrix
Tregs	Regulatory T cells
