# Supplementary material for: Tumor-Infiltrating Immune Cell Landscapes in the Lymph Node Metastasis of Papillary Thyroid Cancer
Source: Curr Oncol. 2023 Feb 22;30(3):2625–41. doi: 10.3390/curroncol30030200 (PMC10046895; doi:10.3390/curroncol30030200)
Supplement: Supplementary file 1 [file curroncol-30-00200-s001.zip › curroncol-2194153-supplementary.pdf]

Tumor-Infiltrating Immune Cell Landscapes in the Lymph Node Metastasis of Papillary Thyroid Cancer

Md Amanullah et al.

Supplementary Materials

Table S1 Descriptive **s**ummary of thyroid carcinoma patients with and without LNM

**Feature**	**Number**	**Feature**	**Number**
Gender	Male: 90<br>Female: 234	Vital status	Dead: 313<br>Alive: 11
Day to last follow up	Median: 957<br>Min: 32<br>Max: 5150	Age	Median: 46<br>Min: 15<br>Max: 88
AJCC pathologic stage	I: 181; II: 34<br>III: 78; IVA: 26<br>IVC: 5	AJCC pathologic M	M0: 316<br>M1: 8
AJCC pathologic T	T1: 32; T1a: 15<br>T1b: 44; T2: 99<br>T3: 117; T4a: 10<br>Other: 7	AJCC pathologic N	N0: 170<br>N1: 57<br>N1a: 56<br>N1b: 41
Tissues	Tumor: 283<br>Normal: 58	Metastasis	LNM: 131<br>nLNM: 145

Note:
LNM (lymph-node metastasis): T1-4N1M0; nLNM (non-lymph-node metastasis: T1-4N0M0). Other TCGA PTC specimens that did not belong to these two categories were not included in this study.

Table S2 Differential abundances of immune cells between wild-type and mutant samples

Immune cells	Driver genes	P-value
**Dendritic cells activated**	BRAF	3.15E-05
	APC	4.33E-02
**Eosinophils**	BRAF	7.23E-09
	NRAS	9.33E-05
	HRAS	2.18E-03
	TG	1.90E-02
**Macrophages M0**	BRAF	3.72E-14
	NRAS	1.17E-04
	HRAS	7.85E-03
	PTEN	4.75E-02
	TG	2.21E-03
	EZH1	3.17E-02
**Macrophages M2**	BRAF	1.57E-02
	CHEK2	4.49E-03
	PTEN	3.32E-02
**NK cells Activated**	BRAF	1.72E-02
	PTEN	4.59E-02
	TG	5.62E-03
**T cells regulatory**	BRAF	1.78E-05
	NRAS	1.90E-03
	RB1	2.49E-02
	EZH1	2.13E-02

Note: WT, wild-type; MUT: mutant.

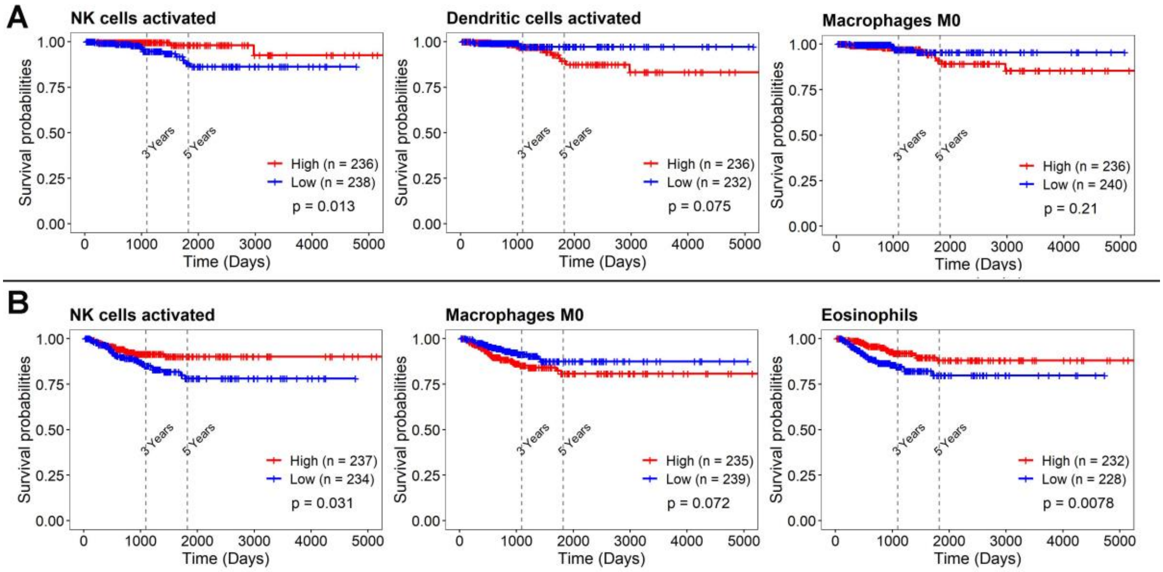

Figure S1. Kaplan-Meier analysis of PTC's survival based on abundances of immune cells. (**A**) Overall survival (OS). (**B**) Progression-free survival (PFS). PTC patients were divided into two groups according to their median levels of immune cell abundance. Difference in survival time between two groups was assessed using a log-rank test. The red and blue colors indicate the high and low abundance of immune cells, respectively. The two vertical gray dashed lines indicate the patient's 3-year and 5-year survival times, respectively. PTC samples with P-values >0.05 were excluded based on CIBERSORTx estimates of tumor-infiltrating immune cell abundance, as their immune cell composition was not considered reliably estimated. Therefore, different numbers of PTCs were able to be analyzed in each KM analysis.

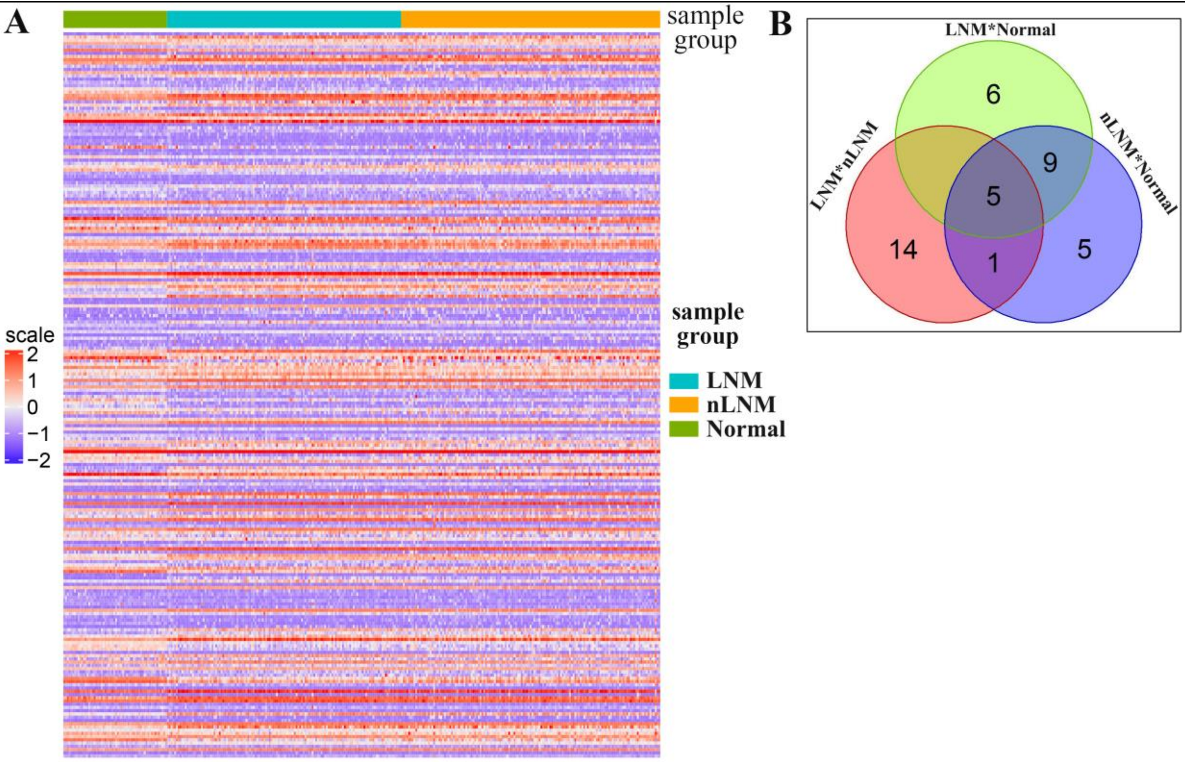

Figure S2. Heatmap of differentially expressed genes between PTC groups. **(A)** The heatmap shows the expression patterns of 300 highly DEGs in three groups (PTC tumors with and without LNM, and adjacent normal tissues), of which 100 highly DEGs were selected from each pair of comparisons. Each column represents a sample, and each row represents a gene. The expression level of each gene in a single sample is depicted according to the color scale. **(B)** Venn diagram showing the number of highly enriched pathways overlapping between the three pairs of comparisons, of which 20 highly enriched pathways were selected from each comparison pair.

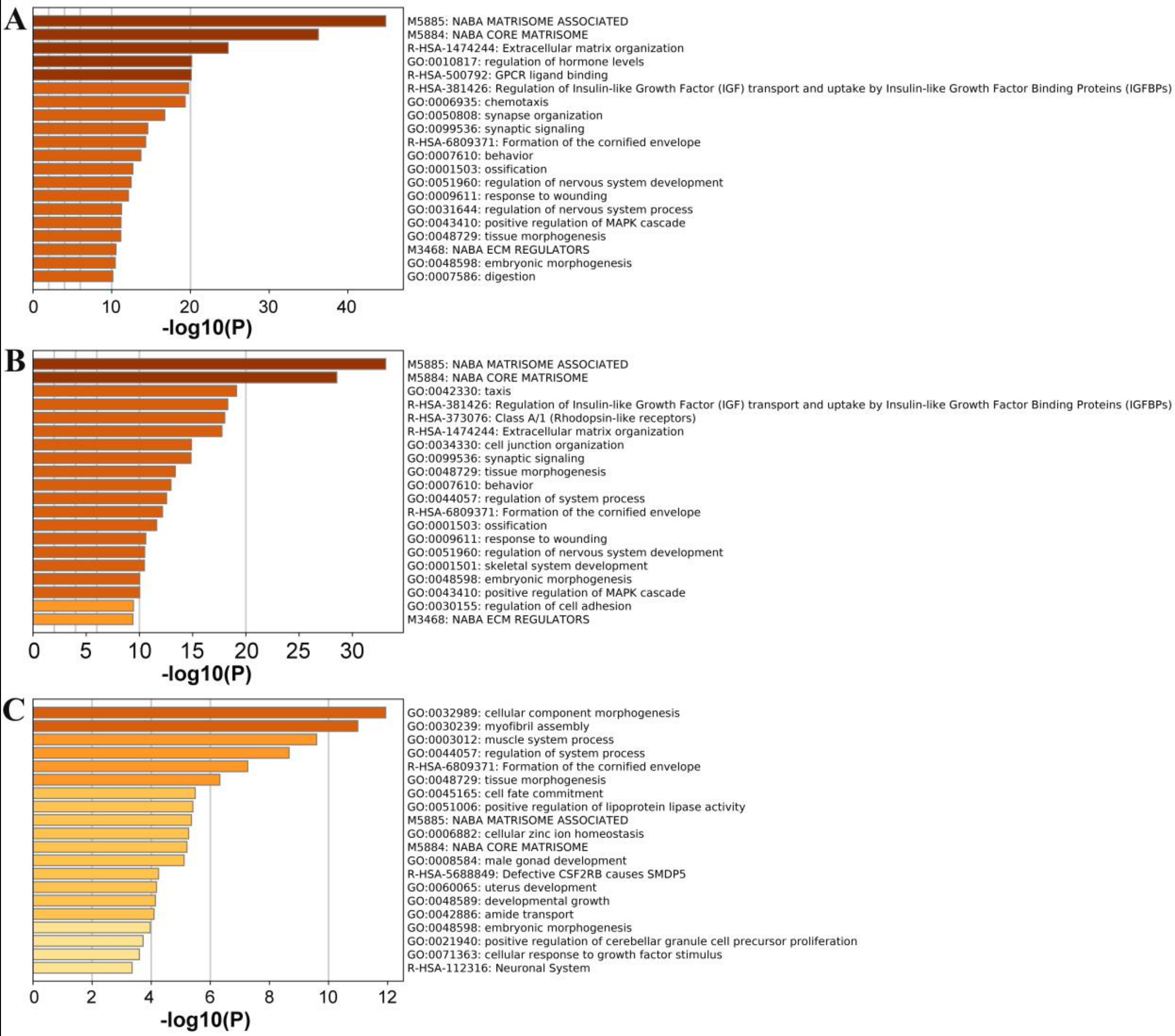

Figure S3. Functional enrichment analysis of differentially expressed genes between PTC groups. **(A)** Top 20 GO and KEGG pathways enriched for DEGs between LNM and normal. **(B)** Top 20 GO and KEGG pathways enriched for DEGs between nLNM and normal. **(C)** Top 20 GO and KEGG pathways enriched for DEGs between LNM and nLNM. Enrichment analysis was performed using the Metascape portal. The x-axis corresponds to -log10 *P*-values of enrichment, whilst the y-axis indicates enriched pathways. The color density indicates the significance level of a given pathway.

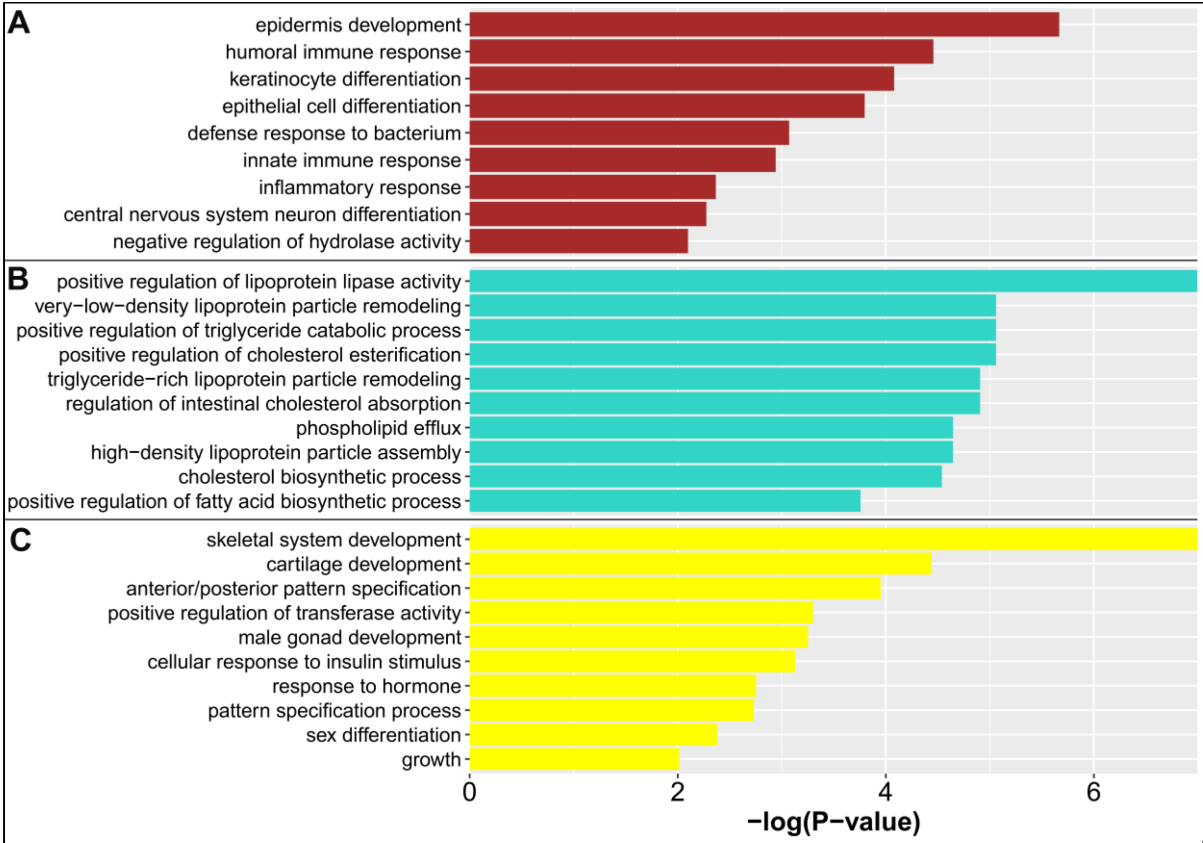

Figure S4. GO and pathway enrichment analysis of three gene co-expression modules. Enrichment analysis of GO terms and pathways conducted for the gene modules correlated with survival-related immune cell subsets. **(A)** Brown module that showed the highest positive correlation with dendritic cells activated, and the highest negative correlation with eosinophils. **(B)** Turquoise module that was positively correlated with the abundance of eosinophils. **(C)** Yellow module that was positively correlated with M0 macrophages and active dendritic cells and negatively correlated with activated NK cells and eosinophils. The x-axis corresponds to -log10 *P*-values of enrichment, while the y-axis indicates enriched GO and pathways.
